# Connected Health Devices for Health Care in French General Medicine Practice: Cross-Sectional Study

**DOI:** 10.2196/mhealth.7427

**Published:** 2017-12-21

**Authors:** Leila El Amrani, Agnes Oude Engberink, Gregory Ninot, Maurice Hayot, François Carbonnel

**Affiliations:** ^1^ UFR Medecine site Nord Department of General Practice University of Montpellier Montpellier France; ^2^ Centre Hospitalier de Carcassonne Department of Emergency Medicine Carcassonne France; ^3^ CEPS Platform Universities of Montpellier Montpellier France; ^4^ Research Unit EA4556 Epsylon University of Montpellier Paul Valery University of Montpellier Montpellier France; ^5^ Avicenne Multiprofessional Health Center Cabestany France; ^6^ Institut du Cancer Montpellier Montpellier France; ^7^ PhyMedExp, University of Montpellier CNRS, INSERM CHU Montpellier Montpellier France

**Keywords:** general practice, physician-patient relations, monitoring, ambulatory, wireless technology, medical informatics, telemedicine

## Abstract

**Background:**

The integration of Connected Health Devices (CHDs) is growing within mobile health (mHealth) and telemedicine, encouraged by institutions and industries. The idea is to improve lifestyle habits and health behaviors as a preventive goal in an aging population with fewer physicians available. However, their ill-defined place in health care does not promote their use in current medical practice.

**Objective:**

The primary objective of this study was to quantify CHDs’ use rate by general practitioners (GPs). A secondary objective was to evaluate their benefits and limitations in usual care.

**Methods:**

A cross-sectional study through an Internet-based survey was addressed to French GPs via regional medical unions and continuous education agencies, supplemented with an informative website, from March 2015 to July 2015. Surveys where either the form was insufficiently filled or the main question was left unanswered were excluded from the study.

**Results:**

A total of 1084 answers were analyzed, of which 19.46% (211/1084, 95% CI 17.1-21.8) GPs used CHDs, and 10.15% (110/1084, 95% CI 8.5-12.1) prescribed a CHD. CHD users statistically prescribed more CHDs (7.38% [80/1084] in the user group vs 2.86% [31/1084] in nonusers; *P*<.001) and were more likely to use them in the future. Major interests in their utilization were in patient monitoring for 84.96% (921/1084) and patient education for 75.83% (822/1084), especially for diabetes (89.67%, 972/1084) and hypertension (84.13%, 912/1084). Generated data had to be managed securely by the patient primarily for 85.79% (930/1084) of the GPs. CHDs had to not constrain GPs outside clinical consultation, nor restrain their time for 75.83% (822/1084). Additional actors in patient care were not desired for 79.98% (867/1084) of the GPs. Questions about data management issues and technical difficulties were raised.

**Conclusions:**

CHDs are little used by French GPs and even less prescribed to their patients, as only a few GPs use these tools. Their benefits as tools of patient empowerment, although expected, remain to be demonstrated in real-life setups.

## Introduction

Mobile health (mHealth) is the medical and public health practice supported by mobile devices such as smartphones, patient monitoring devices, personal digital assistants, and other wireless devices (World Health Organization, WHO) [1]. Two major categories of mHealth tools are now being developed: mobile phone apps and connected health devices (CHDs). CHDs are objects generating physiologic information synchronized via a wireless network (Wi-Fi or Bluetooth) to a mobile phone, tablet, manufacturer’s website, and so on. They make it possible to get rid of pen and paper and to access processed information—from numbered data to graphic reports, or to alarms of exceeded threshold. They communicate with other computer systems to obtain or provide additional information: these are called *intelligent* objects. They may be objects intended for medical use or often objects for managing health and well-being.

By the end of 2016, major app stores listed more than 259,000 mHealth apps and reached 3.2 billion downloads [2]. Economic impact of CHDs and connected fitness was about 190 billion US dollars worldwide and is expected to double by 2020 [3].

Technological advancements in CHDs have allowed the development of the self-quantification of a wide range of aspects of a person’s life, such as quality of sleep, physical activity, blood pressure, or mental state, as well as the blooming of trending behaviors such as the quantified self. According to the WHO, "up to 80% of cardiovascular diseases (...) and more than a third of cancers could be avoided by eliminating risk factors such as (...) sedentary behaviors" [4], and those could account for 40% of premature deaths in the United States [5].

Treatment adherence in chronic diseases is a key element as, “increasing the effectiveness of adherence interventions may have a far greater impact on the health of the population than any improvement in specific medical treatments” [6]. Surveys reported real expectations of health professionals, patients, and industrials about CHDs [7]. Some studies showed an improved [8], longer-lasting [9], hypertension control achieved through remote monitoring. A 2015 meta-analysis of randomized controlled trials evaluating remote monitoring in heart failure showed a significant reduction in mortality (odds ratio [OR]=0.52, 95% CI 0.37-0.72 compared with usual care) and hospital admissions (OR=0.70, 95% CI 0.51-0.96) [10].

Overall, 69% of US adults keep track of at least one health indicator such as weight, diet, exercise routine, or a symptom, with 21% using a technology-based tracker [11]. Of these, 46% of adults using trackers said that it had changed their overall approach to maintaining their health. For PEW Internet Research Center and according to a study published in *Health Care*, about two-thirds of Americans were monitoring at least one health indicator, 20% of them with a form of technology; however, only half of the general population was sharing these data with their physicians (via email or a screen) [11,12].

General practitioners (GPs) are gatekeepers to accessing care in many health care systems, and so are the GPs in France. They might be an interesting medium to develop CHDs’ use, and they could use CHDs to engage and maintain patients’ behaviors into healthier ones. Little is known about how they integrate CHDs in their toolbox as nonpharmacological interventions. The primary objective of this study was to quantify the current use of CHDs by GPs in France. The secondary objectives were to assess the place of CHDs in patient care in general practice and to identify barriers to their use, according to GPs.

## Methods

**Design**

We conducted a cross-sectional survey using Google forms and emails to GPs (Multimedia Appendix 3). The questionnaire was developed by the authors LEA and FC based on PubMed bibliographic research and data retrieved from European health and economic organizations’ documents. The questionnaire consisted of 25 questions divided into four sections. Section 1 collected data about personal and professional use of CHDs. Section 2 focused on GPs’ patients’ use of CHDs. Section 3 elicited information on their benefits, the barriers to their use, and how to optimize their integration into their daily practice. Section 4 collected demographic data related to the practice and GPs.

A link to a website purposely developed to inform or remind GPs about the different CHDs available worldwide was included in that email (Multimedia Appendix 2). For the purposes of the study, CHDs were classified by the authors as connected medical devices (ie, glucometers, oximeters, sphygmomanometers, pill boxes, thermometers, and peakflow meters), as health-related connected devices in general or “mainstream” CHDs (ie, weight trackers, activity and sleep trackers, breath-analyzers, smoking cessation tools, diet monitors, oral hygiene and prevention of low back pain trackers, trackers for infants and seniors, and tools for pregnancy and quality of air monitoring), and as other specialized connected devices (urine “scanners,” specialized epilepsy clothing, and so on).

The survey remained available on the Web from March 10, 2015 to July 10, 2015. Responses were collected automatically on an Excel (Microsoft) type spreadsheet. Multiple submissions from a single submitter were resolved using Internet protocol addresses and time stamps from submission.

The review board from the department of General Practice of University of Montpellier gave their approval before submitting the survey to the different organisms.

**Population**

This work focused on GPs in France. To reach the GPs, the 26 Regional Health Practitioners Unions (Union Régionale des Professionnels de Santé, URPS) of France were contacted by phone starting March 10, 2015 to collect their email addresses, then by email to present the study and survey for distribution to all GPs registered to them. They were contacted again by email on May 2015 to dispatch the email once more to improve the survey’s distribution. A total of 26 URPS were initially contacted: 11 URPS refused to participate, 15 emailed the questionnaire to the GPs registered on their mailing list, and 9 dispatched the email again on May 2015.

The survey was also spread through the GPs’ “UG-Zapping” newsletter and the continuous medical education association “FMC-Action”. The link to the survey was published in the monthly newsletter “UG-Zapping” on April 6, 2015 and was distributed by email to the 35,000 GPs members of “FMC-Action” in early June 2015.

Exclusion criteria were lack of response to the question meeting the primary objective of the study, or answering less than 80% of the survey.

**Statistical Analysis**

The number of subjects needed to achieve statistical significance with a risk of 5%, based on bibliographic databases, was 384.

An initial descriptive analysis on the included population was performed.

We named the group currently using the CHD “CHD+” and the nonuser group “CHD−.”

Qualitative variables were expressed by their effectives and their percentage (number, %). Comparison of qualitative variables was executed through the chi-square test for parametric tests, or Fisher exact test when the conditions for applying chi-square were not observed, and performed on Microsoft Excel and BiostaTGV software. The significance threshold was set at 5%.

## Results

### Population Characteristics

A total of 1086 GPs from all over France responded. Of the 1086 GPs who answered the questionnaire, 1084 were included in the analysis, as two of the responders met the exclusion criteria.

Demographics of GPs who responded to the survey are shown in Table 1.

**Personal and Professional Use of Connected Health Devices**

Of the 1084 GPs included, 211 were using a CHD at the time of the study (“CHD+”; ie, 19.46%; 95% CI 17.1-21.8) versus 873 GPs who were not using a CHD (“CHD−”; ie, 80.54%; 95% CI 78.2-82.9).

Among the CHD+, 105 were using a connected device for general health or “mainstream” (ie, 49.8%, 105/211; 95% CI 7.9-11.4), 70 a connected medical device (ie, 33.2%, 70/211; 95% CI 5-7.9), and 36 used both (ie, 17%, 36/211; 95% CI 2.3-4.4). CHDs most used by GPs were connected glucometers in first place (29.9%, 63/211 of users), activity trackers (18.5%, 39/211 of users), and connected sphygmomanometers (17.5%, 37/211); 51.7% (109/211) of users did not answer the previous question.

A total of 111 GPs had prescribed CHDs to their patients (10.15%, 110/1084; 95% CI 8.4-12.0) versus 971 who had not (ie, 89.57%, 971/1084; 95% CI 87.8-91.4). Physicians using connected devices significantly prescribed CHDs more than those who were not (7.38%, 80/1084 in the “CHD+” group vs 2.86%, 31/1084 in the group “CHD−“; *P*<.001). There was no statistical difference between prescription of “mainstream” CHDs and those for medical purposes (respectively 4.43%, 48/1084 vs 3.97%, 43/1084; *P*=.549).

**Figure 1:**
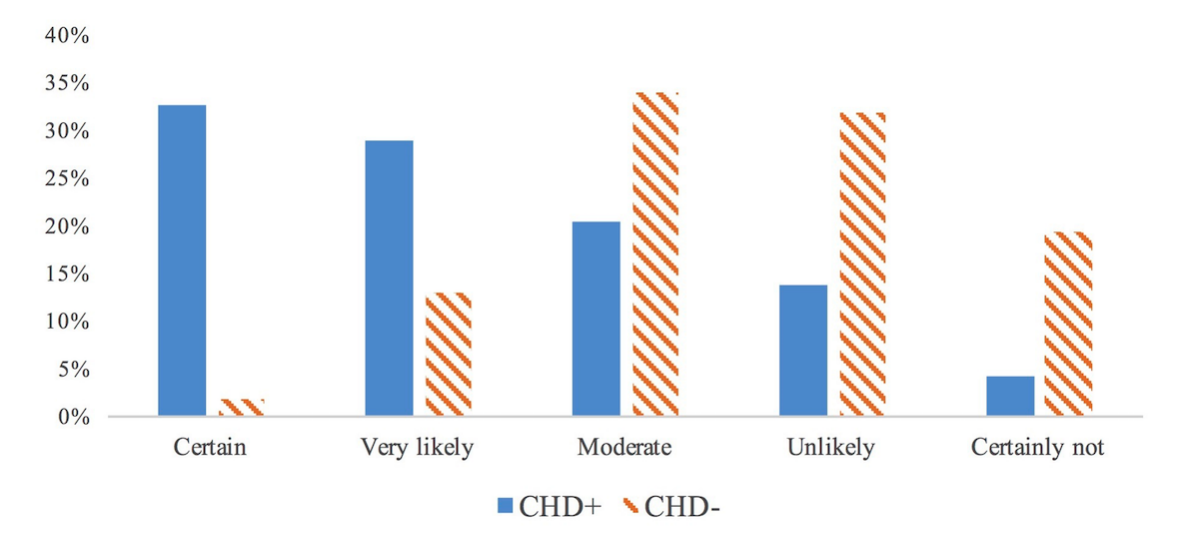
Probability of CHD (connected health device) use in 5 years (n=1084).

In the near future, physicians-CHD users thought “very likely” (28.9%, 61/211 of them) or “certain” (32.7%, 69/211 of them) to use such objects in 2020 (Figure 1). On the contrary, GPs non-CHD users thought that the likelihood of using such items in the future was “unlikely” (31.9%, 278/873 of them) or “moderate” (33.9%, 296/873 of them).

### Patients’ Use of CHDs

A total of 428 GPs (39.48%, 428/1084) were aware of their patients using CHDs; 11.72% (127/1084) of GPs received their patients’ CHDs’ data (vs 88.28%, 957/1084 who did not receive them). They received it either through email, secured messaging, a secured website, or through “other” means.

Patients often shared general health information (in whatever mode) with their GP: 855 (78.87%) of the responding physicians were receiving numbered health data from their patients, with no difference between the two groups (*P*=.93). They received these data primarily on paper records (n=816, 95.4%).

Additionally, 652 (60.2% of responders) were interested in automating these health data, especially in the “CHD+” group (80.1% [169/211] in the group “CHD+” vs 55.3% [483/873] in the group “CHD−”; *P*<.001). There was a link between the interest in data automation and the fact that physicians were already receiving numbered data from their patients: GPs whose patients shared numbered health data (in whatever mode) were more interested in data automation (50.00%, 542/1084 vs 10.33%, 112/1084; *P*<.001).

### Benefits of CHDs in Patients’ Care

GPs generally recognized a usefulness to CHDs for their patients. They felt they could provide assistance in some chronic diseases (Table 2).

They could also be an aid in the GPs’ patient’ management: follow-up, health education, and therapeutic education; however, 3.2% of the responding doctors thought they had no use (Table 2).

Now or in the near future, these tools could enable early intervention, prevent crisis management of a pathology, and avoid disease progression and complications (for 62.55%, 678/1084). For the CHD+ group, they also could help reduce hospital admission or readmission, health care costs, and improve quality of life (Table 3).

**Table 1:** Comparison of general practitioners’ demographics (N=1084); some general practitioners chose not to answer some questions; hence, data can be missing, and totals are not all 100%.

Group		CHD^a^+ (N=211) n (%)	CHD− (N=873) n (%)	*P* value	Total (N=1084) n (%)
**Sex**					
	Female	67 (31.8)	381 (43.6)	.002	448 (41.33)
	Male	139 (65.9)	479 (54.9)		618 (57.01)	
**Age (years)**					
	Less than 35	23 (10.9)	122 (14.0)	.61	145 (13.38)
	36-50	72 (34.)	282 (32.3)		354 (32.66)
	51-65	104 (49.)	426 (48.8)		530 (48.89)
	More than 65	12 (5.7)	41 (4.7)		52 (4.80)
**Smartphone, tablet, or smartphone and tablet use**					
	Yes	205 (97.2)	702 (80.4)	<.001	907 (83.67)
	No	5 (2.4)	170 (19.5)		175 (16.14)
**Smartphone and tablet model type**					
	iPhone	132 (62.6)	365 (41.8)	.18	497 (45.85)
	Android mobile phone	68 (32.2)	263 (30.1)		331 (30.54)
	Windows mobile phone	3 (1.4)	27 (3.1)		30 (2.77)
	Blackberry	5 (2.4)	5 (0.6)		10 (0.92)
	iPad	107 (50.7)	269 (30.8)		376 (34.69)
	Android tablet	37 (17.5)	82 (9.4)		119 (10.98)
	Windows tablet	9 (4.3)	18 (2.1)		27 (2.49)
**Type of practice**					
	Private practice	181 (85.8)	743 (85.1)	.29	924 (85.24)
	Employee	8 (3.8)	18 (2.1)		26 (2.40)
	Substitute	7 (3.3)	46 (5.3)		53 (4.89)
	Mixed	10 (4.7)	33 (3.8)		43 (3.97)
**Teaching position (professor, clinical assistant, or lecturer)**					
	Yes	49 (23.2)	146 (16.7)	.03	195 (17.99)
	No	162 (76.8)	721 (82.6)		883 (81.46)

^a^CHDs: connected health devices.

**Table 2:** Pathologies in which connected health devices (CHDs) could be useful according to general practitioners (GPs; N=1084).

Total size (%)		Yes, n (%)	95% CI	No, n (%)	Unknown, n (%)
**In pathologies (N=1084)**					
	Hypertension	912 (84.13)	82-86.3	101 (9.32)	71 (6.55)
	Diabetes	972 (89.67)	87.9-91.5	58 (5.35)	54 (4.98)
	Obesity	494 (45.57)	42.6-48.5	354 (32.66)	236 (21.77)
	Chronic obstructive pulmonary disease	528 (48.71)	45.7-51.7	292 (26.94)	264 (24.35)
	Asthma	642 (59.23)	56.3-62.2	221 (20.39)	221 (20.39)
	Sleep apnea	643 (59.32)	56.4-62.2	230 (21.22)	211 (19.47)
	Elderly falls	461 (42.53)	39.6-45.5	300 (27.68)	323 (29.80)
	Health (as defined by the WHO^a^)	284 (26.20)	23.6-28.8	350 (32.29)	450 (41.51)
**In situations (N=1084)**					
	Aid to follow-up	921 (84.96)	82.8-87.1	87 (8.03)	76 (7.01)
	Aid adapting treatment	849 (78.32)	75.9-80.8	135 (12.45)	100 (9.23)
	Access to recorded data	832 (76.75)	74.2-79.3	153 (14.11)	99 (9.13)
	Health education	822 (75.83)	73.3-78.4	119 (10.98)	143 (13.19)
	Treatment education	817 (75.37)	72.8-77.9	113 (10.42)	154 (14.20)
	Patient empowerment	702 (64.76)	61.9-67.6	139 (12.83)	243 (22.42)
	Help addressing to a specialist	451 (41.61)	38.7-44.5	442 (40.77)	191 (17.62)
	No use	35 (3.23)	2.2-4.3	437 (40.31)	612 (56.46)
	Help achieving a diagnosis	455 (42.0)	39-44.9	405 (37.36)	224 (20.66)
	Help prescribing additional tests	481 (44.4)	41.4-47.3	396 (36.53)	207 (19.10)
	No use	35 (3.23)	2.2-4.3	437 (40.31)	612 (56.46)

^a^WHO: World Health Organization.

**Table 3:** Current or future usefulness of connected health devices (CHD) according to general practitioners (GPs; N=1084).

Total size (%)		CHD^a^+ (N=211) n (%)	CHD− (N=873) n (%)	*P* value	Total (N=1084) n (%)
**Enable an early intervention**					
	Yes	168 (79.6)	584 (66.9)	.002	752 (69.37)
	No	23 (10.9)	151 (17.3)		174 (16.05)
	Unknown	20 (9.5)	138 (15.8)		158 (14.58)
**Enable crises prevention**					
	Yes	149 (70.6)	490 (56.1)	<.001	639 (58.95)	
	No	35 (16.6)	194 (22.2)		229 (21.13)
	Unknown	27 (12.8)	189 (21.6)		216 (19.93)
**Avoid disease progression and complications**					
	Yes	151 (71.6)	527 (60.4)	.01	678 (62.55)
	No	32 (15.2)	189 (21.6)		221 (20.39)
	Unknown	28 (13.3)	157 (18.0)		185 (17.07)
**Reduction of medical interventions**					
	Yes	81 (38.4)	233 (26.7)	<.001	314 (28.97)
	No	101 (47.9)	458 (52.5)		559 (51.57)
	Unknown	29 (13.7)	182 (20.8)		211 (19.47)
**Reduction of hospital admission**					
	Yes	106 (50.2)	291 (33.3)	<.001	397 (36.62)
	No	67 (31.8)	378 (43.3)		445 (41.05)
	Unknown	38 (18.0)	204 (23.4)		242 (22.32)
**Reduction of hospital readmission**					
	Yes	108 (51.2)	299 (34.2)	<.001	407 (37.55)
	No	61 (28.9)	347 (39.7)		408 (37.63)
	Unknown	42 (19.9)	227 (26.0)		269 (24.82)
**Quality of life improvement**					
	Yes	133 (63.0)	378 (43.3)	<.001	511 (47.14)
	No	34 (16.1)	227 (26.0)		261 (24.08)
	Unknown	44 (20.9)	268 (30.7)		312 (28.78)
**Reduction of health costs**					
	Yes	105 (49.8)	294 (33.7)	<.001	399 (36.81)
	No	45 (21.3)	275 (31.5)		319 (29.43)
	Unknown	61 (28.9)	304 (34.8)		365 (33.67)
**Reduction of mortality**					
	Yes	83 (39.3)	233 (26.7)	.002	316 (29.15)
	No	60 (28.4)	279 (32.0)		339 (31.27)
	Unknown	68 (32.2)	361 (41.4)		429 (39.58)

^a^CHDs: connected health devices.

**Table 4:** Recipients of connected health devices (CHD)–generated data according to general practitioners (GPs; N=1084).

Total size, n (%)	Yes, n (%)	95% CI	No, n (%)	Unknown, n (%)
Patient	930 (85.79)	83.7-87.9	83 (7.66)	71 (6.55)
General practitioner	796 (73.43)	70.8-76.1	159 (14.67)	129 (11.90)
Specialist from the field of the data	699 (64.48)	61.6-67.3	211 (19.47)	174 (16.05)
Patient's nurse	651 (60.06)	57.1-63	278 (25.65)	155 (14.30)
Patient's private supplementary insurance	16 (1.48)	0.8-2.2	974 (89.85)	94 (8.67)
Patient's state insurance	24 (2.21)	1.3-3.1	962 (88.75)	98 (9.04)
Private company	43 (3.97)	2.8-5.1	918 (84.69)	123 (11.35)
Patient's pharmacist	174 (16.05)	13.9-18.2	715 (65.96)	195 (17.99)
Health network	414 (38.19)	35.3-41.1	475 (43.82)	195 (17.99)
Other physical or moral people	514 (47.41)	44.4-50.4	369 (34.04)	201 (18.54)
Patient's helper	20 (1.85)	1-2.6	350 (32.29)	714 (65.87)

### Barriers to the Use of CHDs in Patients’ Care

Main barriers were the data generated themselves and how they could generate anxiety for the patients. They might be generated in excessive quantities, lead to problems in their analysis, be too time-consuming to be used during or outside clinical consultation, or to learn how they work (Multimedia Appendix 1). Regarding data security, GPs were divided: for 482 (44.46%, 482/1084) of the responders, the lack of data security was a hindrance to their use, against 441 (40.68%, 441/1084) who considered that this did not preclude using them (*P*=.07).

GPs would agree to receiving these data in the form of a graphic synthesis (78.32%, 849/1084) but would not agree to receiving them in the form of raw data (68.54%, 743/1084). They were divided on the fact of receiving these data in the form of automatic alerts (48.06%, 521/1084 would accept vs 38.56%, 418/1084 who would not) or interpreted by another health professional (32.47%, 352/1084 would accept vs 44.28%, 480/1084 who would not, and 23.25% (252/1084) were undecided); 7.56% (82/1084) would rather not receive any data from these devices, especially in the nonuser group (2.8%, 6/211 in the group “CHD+” vs 8.7%, 76/873 in the group “CHD−”; *P*=.04).

They also were worried how these CHDs and generated data would affect patient-doctor communication. A total of 539 (49.72%, 539/1084) were worried of legal responsibility of data; 42.25% (458/1084) considered that they should not be constrained by a delay of response to the alert. The appropriate time to respond to the automatic alert would be in the half-day (16.61%, 180/1084) or in the day (17.99%, 195/1084). They considered that the appropriate time for data interpretation per day should not exceed 10 minutes (53.04%, 575/1084). About a third of them, however, considered that the interpretation of these data should not hold any of their daily time (31.09%, 337/1084), especially for CHD nonusers (33.9%, 296/873 in the group “CHD−” vs 19.4%, 41/211 in the group “CHD+”; *P*<.001).

Mostly, this automatic alert should take the form of an email (32.93%, 357/1084), with no difference between groups (*P*=.12).

Regarding data management, the data generated should mainly be received by the patient himself (85.80%, 930/1084; Table 4).

Technical problems such as the lack of interoperability between devices themselves and with medical software were also raised, especially for the “CHD+” group (*P*=.001).

GPs were also concerned by the costs induced by their use for the physicians (no compensation for their use) and for the patients (lack of reimbursement of CHDs and the high cost of these devices; Multimedia Appendix 1).

Other obstacles to their use were identified in the “CHD−” group: absence of recommendation by scientific societies (53.0%, 463/873 of them, *P*=.03) and time GPs must invest in learning how to use them (56.9%, 497/873 of them, *P*<.001).

## Discussion

### Principal Findings

Only 19.47% (211/1084) of the interviewed French GPs were using CHDs and 10.15% (110/1084) prescribing them to their patients.

The users already are in an innovative environment. They have a teaching position, which fosters exchanges with younger students who are widely exposed to Web technologies [13]. They have adopted mHealth tools such as smartphones or tablets [14]. Thus, they were more keen on believing that the CHDs would be a part of their 2020’s toolbox, and they even prescribed more CHDs than nonusers. These results indicate that CHDs are compelling and that CHDs’ path to success in health will probably amplify with the labor market entry of “millennial” medical students.

For the moment, most used CHDs are in fact well-known medical devices—such as glucometers and sphygmomanometers —upgraded to be connected to a wireless device. As for the popularity of physical activity trackers, it is probably related to the widespread notion of a WHO recommendation to take 10,000 steps a day recorded by a pedometer [15].

Although their use is widespread, GPs are falling behind [16]: 39.5% of GPs were aware of their patients using CHDs, which was twice the GP’s use rate. This suggests that GPs are not initiators of the CHD spread. These results are not surprising as these technologies were made to empower patients and are patient-centered.

However, there are wide possibilities to integrate CHDs in medical practice. For example, almost 80% (867/1084) of GPs declared that they received numbered health data from their patients, almost systematically on paper. Interest for data automation was particularly strong among the GPs who already shared automated data with their patients. CHDs might be used to monitor chronic diseases [17] and prevent crisis management of a pathology or its complications. CHD users went further into believing that these tools could help reduce hospital admission, readmission, and health care costs, as well as improve quality of life. Those beliefs are in line with the goals of these objects’ industrial design, as well as those of Public Health, facing an aging population with chronic diseases in the context of a reduced number of physicians and budgetary constraints [18]. The European Commission believes that “mHealth could (...) promote the transition to a preventive approach while increasing the efficiency of the system” and that “remote monitoring using mobile health solutions could decrease by about 15% the cost of healthcare” [7].

Still, if current clinical research studies highlighted improvements in morbidity, mortality, and results in chronic diseases [8,10,19] with the use of connected objects, they have not yet shown such results just by themselves. Each and every time, CHDs were teamed with the intervention of partners to the patients, such as a trained nurse or the intervention of new health actors such as specialized companies. A 2013 study found no benefit on mortality in heart failure with remote monitoring through a human to machine interface [20]. A meta-analysis about educative telemonitoring of decompensated heart failure showed an insufficient or a low level of evidence in hospital readmissions and mortality up to a 6-month follow-up [21]. Improvement to one’s health could be not only linked to the use of connected devices but to the debriefing of their, even irregular, results [22].

GPs identified the following barriers to CHD use: CHDs themselves and their impact on GPs, on their patients, and on doctor-patient relationship.

Concerns expressed by CHD users were mainly focused on technical aspects such as interoperability issues with the software already used by GPs. Implementation of new technologies with their technical challenges create a risk of a digital divide already widely noticed [23,24]. This echoes the delays and technical difficulties known in France with the deployment of the electronic medical records. CHD reliability was also questioned—as devices and as software—particularly by CHD users. Few connected objects are in fact approved by regulatory authorities. Until 2014, the Food and Drug Administration (FDA) had only approved iHealth’s balance and oximeter, Withings’ and Qardio’s sphygmomanometers, AliveCor’s EKG, and Propeller Health’s spirometer [25]. The FDA has decided to intervene only when their use was considered a risk for the patients. European regulatory authorities have, for their part, not yet issued specific recommendations. Both users, and in particular nonusers, agreed that CHDs may create new time constraints to GPs because of the time needed to learning and teaching patients how to use them and analyze data.

How the data generated by CHDs was integrated to the patient-doctor communication was a concern expressed par GPs, mainly by nonusers. They agreed that it should take the form of an email alert. But there were neither agreements on the appropriate response time to the alerts nor on how these data should be treated: whether automated via automatic alerts or interpreted by another health professional. They only agreed on the fact that they did not want these data to be raw but synthetic as they feared excessive data generation and how time-consuming they might be. It must be recalled that, in France, GPs are still on fee for service. To this date, there are no fees scheduled for CHD management by GPs, and French telemedicine’s fee has just been published at the Official Gazette [26]. Similarly, the cost of CHDs for the patients is questioned by GPs, as in France, efficient medical devices and drugs may be reimbursed by health insurance.

The issue of data security was also raised but, surprisingly, did not worry the population of GPs studied as much as one would expect. Data security should be taken more seriously. The rapid market flow of connected devices still provides them with security vulnerabilities accessible to malware that can be used for larger cyberattacks [27].

Finally, GPs feared that the data generated might cause anxiety or depression [8]. There are also risks of higher performance research [28] and risks of overmedicalization of general population with the spread of devices quantifying various corporeal data as they might become “an integral part of a pervasive, ubiquitous future, of a patient-centered care system” [23]. In addition, there’s a patient safety issue with data self-management by patients themselves “when one can use the results from a device or mobile health application to take himself a decision that might jeopardize his health, or when the information received from the application incorrectly indicates that the person is in good health” [7].

Still, for GPs surveyed, it was the patients that should receive the data generated by the connected objects in priority. Studies emphasized the importance of patients’ education and self-management on their illness to improve adherence and outcomes [29]. This is also the wish of regulators: “one of the main objectives is to enable people to become, through information and communication technologies (ICT), co-managers of their health and well-being” [7]. It was probably to assist them at best that GPs defined themselves and the patient’s nurse as corecipient of the data generated by CHD.

### Strengths and Limitations

Sociodemographic data of GPs in our study were comparable with those of the French health ministry in January 2015 [30]: mean age of 53 years, 55% male, 58% liberal, and 84% equipped with smartphones and/or tablets. Their equipment rate was also similar to other studies administered online to GPs [31]. Lack of links of interest or funding for the study using this type of free distribution, coupled with the anonymity of responses, allowed us to receive honest answers, close to the reality of current medical practice. Emails enabled us to reach a large sample size to improve the power of our study with a more than adequate number of responses but also a diverse sample including GPs working in both urban and rural areas.

However, the way our questionnaire was delivered (many dispatchers and a newsletter) made it hard to calculate a response rate (which would be lower than 3%) in a population knowingly lacking availability even to address issues concerning them [32]. Our nonrandomized study was also exposed to the known selection bias of email surveys: those most interested and those most worried about the subject studied answer more frequently, raising the question about the representativeness of the participating GPs. In addition, our subject was perhaps a bit long to respond to (about 10 minutes), and only the completed questionnaires were saved, probably adding an information and a recall bias. Moreover, GPs may hold little interest in connected objects in their professional practice: the URPS from the Provence Alpes Côte d’Azur region, for example, judged unnecessary to distribute the questionnaire to GPs, as “connected health devices did not match the current practice of medicine.” This refusal might also be explained by their wish to limit the emailed solicitations of the GPs.

### Perspectives

Expectations are high about CHDs for patients, caregivers, and health authorities in particular to prevent and manage chronic diseases, with a broad road ahead of them. mHealth, as electronic health and, in general, nonpharmacological interventions need new ways to be explored and validated to be taken seriously by scientific communities [33]. So far, CHDs’ scientific evaluation meets a reality constraint because of their rapid obsolescence.

Ultimately, for the GPs, CHDs are mostly tools of accountability and patients’ empowerment. They are complementary to the practice of the medical art, which must remain primarily humanist and based on the customized clinical doctor-patient relationship. In the short to medium term, their challenge is to become part of the physician’s toolbox to prevent and manage diseases. As well as being effective and then covered by health insurance, they need to be usable tools created in a “plug and care or cure” philosophy. In a more ambitious step, CHDs might also release themselves from the usual constraints of the doctor-patient relationship and attain their autonomy in health, but integrative and comprehensive CHDs are not marketed yet.

## Appendix

Multimedia Appendix 1 Obstacles to connected health devices’ (CHDs) use according to general practitioners (GPs; N=1084).

Multimedia Appendix 2 Screenshots of the website.

Multimedia Appendix 3 Questionnaire.

## Abbreviations

CHDs: connected health devices
GPs: general practitioners
mHealth: mobile health
OR: odds ratio
URPS: Regional Health Practitioners Unions
WHO: World Health Organization
