# Supplementary material for: Connected Health Devices for Health Care in French General Medicine Practice: Cross-Sectional Study
Source: JMIR Mhealth Uhealth. 2017 Dec 21;5(12):e193. doi: 10.2196/mhealth.7427 (PMC5754567; doi:10.2196/mhealth.7427)
Supplement: Multimedia Appendix 1 [file mhealth_v5i12e193_app1.pdf]

Multimedia Appendix 1. Obstacles to connected health devices' (CHDs) use according to general practitioners (GPs; N=1084).

Total size (%)		CHD+ (N=211) n (%)	CHD− (N=873) n (%)	*P* value	Total (N=1084) n (%)
**TECHNICAL DIFFICULTIES:**					
**Lack of data security**					
	Yes	95 (45.0)	387 (44.3)	.5	482 (44.46)
	No	90 (42.7)	351 (40.2)		441 (40.68)
	Unknown	26 (12.3)	135 (15.5)		161 (14.85)
**Lack of technical reliability of CHD**					
	Yes	100 (47.4)	399 (45.7)	.0017	499 (46.03)
	No	83 (39.3)	267 (30.6)		350 (32.29)
	Unknown	28 (13.3)	207 (23.7)		235 (21.68)
**Lack of software reliability**					
	Yes	99 (46.9)	375 (43.0)	.0014	474 (43.73)
	No	83 (39.3)	278 (31.8)		361 (33.30)
	Unknown	29 (13.7)	220 (25.2)		249 (22.97)
**Interoperability issues**					
	Yes	108 (51.2)	388 (44.4)	.0012	496 (45.76)
	No	55 (26.1)	173 (19.8)		228 (21.03)
	Unknown	48 (22.7)	312 (35.7)		360 (33.21)
**DATA MANAGEMENT:**					
**Legal responsibility problem of data**					
	Yes	109 (51.7)	430 (49.3)	.08	539 (49.72)
	No	74 (35.1)	270 (30.9)		344 (31.73)
	Unknown	28 (13.3)	173 (19.8)		201 (18.54)
**Excess data generated**					
	Yes	143 (67.8)	665 (76.2)	.0339	808 (74.53)
	No	45 (21.3)	129 (14.8)		174 (16.05)
	Unknown	23 (10.9)	79 (9.0)		102 (9.41)
**Data analysis problem**					
	Yes	129 (61.1)	634 (72.6)	.0036	763 (70.39)
	No	57 (27.0)	157 (18.0)		214 (19.74)
	Unknown	25 (11.8)	82 (9.4)		107 (9.87)
**TIME CONSTRAINTS:**					
**Too much time spent on CHD during clinical consultation**					
	Yes	138 (65.4)	649 (74.3)	.0139	787 (72.60)
	No	55 (26.1)	152 (17.4)		207 (19.10)
	Unknown	18 (8.5)	72 (8.2)		90 (8.30)

	**Too much time spent on CHD outside clinical consultation**				
	Yes	148 (70.1)	674 (77.2)	.06	822 (75.83)
	No	43 (20.4)	122 (14.0)		165 (15.22)
	Unknown	20 (9.5)	77 (8.8)		97 (8.95)
	**Too much time invested learning how to use CHD**				
	Yes	76 (36.0)	497 (56.9)	<.001	573 (52.86)
	No	114 (54.0)	296 (33.9)		410 (37.82)
	Unknown	21 (10.0)	80 (9.2)		101 (9.32)
**FINANCIAL CONSTRAINTS:**					
	**Lack of remuneration**				
	Yes	136 (64.5)	533 (61.1)	.6	669 (61.72)
	No	55 (26.1)	258 (29.6)		313 (28.87)
	Unknown	20 (9.5)	82 (9.4)		102 (9.41)
	**Lack of CHD reimbursement**				
	Yes	109 (51.7)	473 (54.2)	.78	582 (53.69)
	No	64 (30.3)	246 (28.2)		310 (28.60)
	Unknown	38 (18.0)	154 (17.6)		192 (17.71)
	**High cost of CHD**				
	Yes	148 (70.1)	610 (69.9)	.0263	758 (69.93)
	No	38 (18.0)	109 (12.5)		147 (13.56)
	Unknown	25 (11.8)	154 (17.6)		179 (16.51)
**PATIENTS' QUALITY OF LIFE:**					
	**Patients do not know how to use CHD**				
	Yes	102 (48.3)	464 (53.2)	.19	566 (52.21)
	No	69 (32.7)	231 (26.5)		300 (27.68)
	Unknown	40 (19.0)	178 (20.4)		218 (20.11)
	**Patients could be worried by data generated**				
	Yes	144 (68.2)	672 (77.0)	.0155	816 (75.28)
	No	50 (23.7)	136 (15.6)		186 (17.16)
	Unknown	17 (8.1)	65 (7.4)		82 (7.56)
**MEDICAL REFERENCES:**					
	**Poor objects' hygiene**				
	Yes	42 (19.9)	177 (20.3)	.45	219 (20.20)
	No	127 (60.2)	489 (56.0)		616 (56.83)
	Unknown	42 (19.9)	207 (23.7)		249 (22.97)
	**Lack of medical reference**				
	Yes	94 (44.5)	463 (53.0)	.0254	557 (51.38)
	No	81 (38.4)	253 (29.0)		334 (30.81)
	Unknown	36 (17.1)	157 (18.0)		193 (17.80)
