# Supplementary material for: Connected Health Devices for Health Care in French General Medicine Practice: Cross-Sectional Study
Source: JMIR Mhealth Uhealth. 2017 Dec 21;5(12):e193. doi: 10.2196/mhealth.7427 (PMC5754567; doi:10.2196/mhealth.7427)
Supplement: Multimedia Appendix 2 [file mhealth_v5i12e193_app2.pdf]

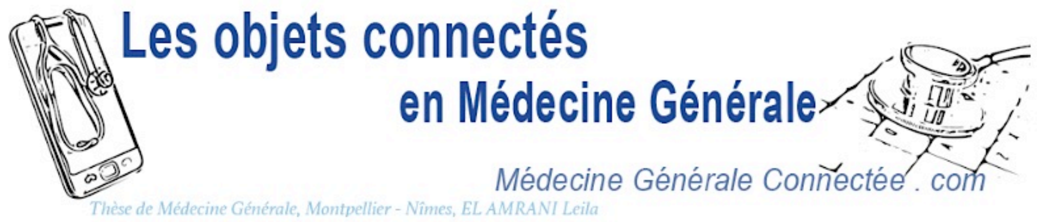

Les objets connectés

Ce sont des objets synchronisés au smartphone, à la tablette, à un site du constructeur.. qui permettent de s'affranchir du carnet papier - stylo, d'avoir accès à des données chiffrées brutes et parfois des rapports graphiques (courbes, mini-maxi, ...) ou des alarmes de dépassement d'un seuil prédéfini. Ils peuvent être des objets à vocation de dispositif médical, ou souvent des objets qui permettent de gérer sa santé et son bien-être de manière général.
Les liens ci-dessous vous amènent au site constructeur, ou à un article décrivant l'objet.

- Objets connectés médicaux
- Objets connectés de santé
- Autres objets connectés spécialisés

Vous êtes ici : Accueil ▸ Les objets connectés

© 2015 Le Quantified Self en Médecine Générale

Back to Top

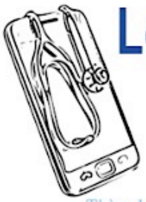

Les objets connectés
en Médecine Générale

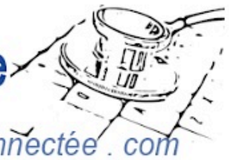

Médecine Générale Connectée . com

Thèse de Médecine Générale, Montpellier - Nîmes, EL AMRANI Leila

Les objets connectés

Ce sont des objets synchronisés au smartphone, à la tablette, à un site du constructeur.. qui permettent de s'affranchir du carnet papier - stylo, d'avoir accès à des données chiffrées brutes et parfois des rapports graphiques (courbes, mini-maxi, ...) ou des alarmes de dépassement d'un seuil prédéfini. Ils peuvent être des objets à vocation de dispositif médical, ou souvent des objets qui permettent de gérer sa santé et son bien-être de manière général.
Les liens ci-dessous vous amènent au site constructeur, ou à un article décrivant l'objet.

▾ Objets connectés médicaux

▾ Glucomètres

Du carnet de dextros aux données automatiquement synchronisées sur un smartphone / tablette / site web, avec alarmes de dépassement de seuil, courbes graphiques, envoi de données au médecin ou un aidant si le patient le souhaite.

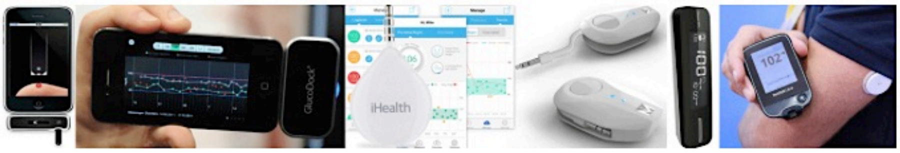

- **Glucomètres 2.0** : iBGStar de Sanofi, iHealth, Glucodock, GlucoLog, Fora Care,
- **Seringue connectée** (Vigipen) : elle permet l'injection d'insuline et la mesure de glycémie capillaire dans le même outil,
- **Patch** (FreeStyleLibre de Abbott) : mesure régulière de la glycémie capillaire à travers le patch
- *Pour en savoir plus...*

▸ Oxymètres

▸ Tensiomètres

▸ Pilulier

▸ Thermomètres

▸ Débitmètre

▸ Canne
