# Supplementary material for: Connected Health Devices for Health Care in French General Medicine Practice: Cross-Sectional Study
Source: JMIR Mhealth Uhealth. 2017 Dec 21;5(12):e193. doi: 10.2196/mhealth.7427 (PMC5754567; doi:10.2196/mhealth.7427)
Supplement: Multimedia Appendix 3 [file mhealth_v5i12e193_app3.pdf]

Multimedia Appendix 3: Questionnaire

Connected Health Devices in General Practice

Thesis of General Medicine, Montpellier-Nimes region:

the Quantified Self in General Practice

To learn more about this study, click on www.mg-connecte.com

1 Before starting the questionnaire ... Do you know what a connected health device is? *(1 choice only)*

○ Yes ○ No

More information on connect devices here: http://www.mgconnectee.com/index.php/wearables

The connected devices are connected blood pressure monitors, connected blood glucose monitors, connected scales ...

They are objects synchronized to a smartphone, a tablet, to the manufacturer's website ... which allow overcoming the paper – pen alliance, having access to raw figures and sometimes graphic reports (curves, minimum-maximum, ...) or alarms of exceeded predefined thresholds. Their purpose can be to be used as a medical device, or often as objects of health management or of well-being in general.

The website www.mg-connectee.com (built for this thesis) can let you know a little more about them.

During this survey, we will differentiate connected health devices from those with medical purpose, we will call them "connected medical devices".

Personal and professional use:

2 Are you currently using at least one of these "connected devices"? *(1 choice only)*

☐ A connected health object ☐ A connected medical object

☐ Both ☐ No

3 Are you prescribing one of these "connected devices"? *(1 choice only)*

☐ A connected health object ☐ A connected medical object

☐ Both ☐ No

If Yes to Question 2 or 3:

Which connected device are you using? (personally or professionally)? *(Multiple choice)*

☐ Activity trackers (Withings Pulse, Polar Loop, Fitbit, Garmin, Smartband, Samsung, Jawbone, Misfit...)

☐ Sleep trackers (Withings Aura, Beddit..)

☐ Smoking cessation trackers (Smokio)

☐ Weight trackers (Withings Smartbody, iHealth, FitBit Aria...)

☐ Connected blood glucose monitors (iBGStar, iHealth, seringue connectée, patch connecté, …)

☐ Connected oximeters (Health, Beurer)

☐ Connected blood pressure monitors (Health, Withings, ...)

☐ Connected pillboxes (Imedipac, iRemember, Medsecure...)

☐ Connected thermometers (Kinsa, e-Celcius)

☐ Connected peakflows (MySpiroo, Propeller Health)

☐ Connected cane (New Generation Cane)

☐ Other: …………

Which 3 connected devices are you using most in your professional practice?

- Free answer

How did you get the connected device? *(Multiple choice)*

☐ Private buy

☐ Private gift

☐ Professional buy

☐ Professional gift (lab, manufacturer, clinical study…)

4 What is the probability of you using a “connected medical device” in your PROFESSIONAL practice in the 5 next years (2020)?

	1	2	3	4	5	
I won’t be using one	○	○	○	○	○	I’m already using one or more of these

<u>Self quantifying in your patients:</u>

5 Is one of your patients currently using at least one "connected device" for medical or health reasons? *(1 choice only)*

☐ A connected health object ☐ A connected medical object

☐ Both ☐ No

<u>If Yes to question 5:</u>

Which devices are your patients using? *(Multiple choice)*

☐ Activity trackers (Withings Pulse, Polar Loop, Fitbit, Garmin, Smartband, Samsung, Jawbone, Misfit...)

☐ Sleep trackers (Withings Aura, Beddit..)

☐ Smoking cessation trackers (Smokio)

☐ Weight trackers (Withings Smartbody, iHealth, FitBit Aria...)

☐ Connected blood glucose monitors (iBGStar, iHealth, seringue connectée, patch connecté, …)

☐ Connected oximeters (Health, Beurer)

☐ Connected blood pressure monitors (Health, Withings, ...)

☐ Connected pillboxes (Imedipac, iRemember, Medsecure...)

☐ Connected thermometers (Kinsa, e-Celcius)

☐ Connected peakflows (MySpiroo, Propeller Health)

☐ Connected cane (New Generation Cane)

☐ Other: …………

6 Are your receiving data from your patients' connected devices?

○ Yes ○ No

<u>If Yes to question 6 :</u>

How do you receive the data? *(Multiple choice)*

☐ Email ☐ Secure emailing

☐ Secure website ☐ Other : …………

7 Are your patients sharing numbered health data through another way than a connected health device? *(for example: blood glucose or blood pressure notebook…)*

○ Yes ○ No

8 Would you be interested in automation of those data?

○ Yes ○ No

If Yes to question 7:

How are your patients sharing those numbered health data? *(Multiple choice)*

☐ Written on paper (table, calendar…)

☐ Written on a computer program (*ie* an Excel spreadsheet)

☐ Through a mobile app

☐ Orally (from memory)

☐ Other : …………

Your opinion on connected medical devices:

9 What are or could be the use of such medical devices in your professional practice? *(1 answer per lign)*

	Yes	No	You don't know
Diagnostic aid for your patient	○	○	○
Follow-up aid of your patient	○	○	○
Patient's health education	○	○	○
Patient's treatment education	○	○	○
Strengthening adherence and "empowerment" of the patient	○	○	○
Access to recorded data of the patient	○	○	○
Decision aid to prescribing biological or radiological complimentary exams	○	○	○
Decision aid to referring your patient to a specialist	○	○	○
Aid to treatment adaptation	○	○	○
None	○	○	○
Other	○	○	○

10 In which pathologies do you think such devices can or could be valuable tools in your PROFESSIONAL practice? *(1 answer per lign)*

	Yes	No	You don't know
Hypertension	○	○	○
Diabetes	○	○	○
Obesity	○	○	○
COPD	○	○	○
Asthma	○	○	○
Sleep apnea	○	○	○
Elderly fall risk	○	○	○
Well being and Health (WHO's definition)	○	○	○
Other	○	○	○

11 Do you think these devices, from here to 2020, could…? *(1 answer per lign)*

	Yes	No	You don't know
Reduce the need of medical interventions	○	○	○
Reduce the need of hospital admissions	○	○	○
Reduce the need for hospital readmissions	○	○	○
Facilitate early interventions	○	○	○
Prevent crisis management	○	○	○
Avoid complications or disease progression	○	○	○
Reduce mortality	○	○	○
Improve quality of life	○	○	○
Reduce health costs	○	○	○

12 What would stop you from using these connected devices with your patients? *(1 answer per lign)*

	Yes	No	You don't know
Lack of security of patients' data	O	O	O
Data liability issue	O	O	O
Lack of technical reliability of these devices	O	O	O
Lack of technical reliability of apps linked to these devices	O	O	O
Poor devices' hygiene	O	O	O
Excess data generated	O	O	O
Data analysis problem	O	O	O
Too much time spent reviewing data during medical consultation	O	O	O
Too much time spent reviewing data outside clinical consultation	O	O	O
Too much time to invest learning how to use these devices	O	O	O
Lack of remuneration of the physician analyzing the data	O	O	O
Lack of inter-operability of devices	O	O	O
Patients don't know how to use the devices	O	O	O
Patients can worry about the results	O	O	O
Lack of patients' reimbursement	O	O	O
Lack of scholarly recommendation	O	O	O
High cost of devices	O	O	O
I don't count on getting one	O	O	O
Other	O	O	O

<u>Your view of connected medical devices:</u>

13 On data analysis from connected medical devices, do you think you should… *(1 answer per lign)*

	Yes	No	You don't know
Receive yourself all raw data	O	O	O
Receive a summary of data in the form of tables or graphical data	O	O	O
Receive automatic alerts in case of an abnormality (for example, blood glucose exceeding a threshold)	O	O	O
Receive data interpreted by another health professional	O	O	O
Other	O	O	O

14 What type of information do you prefer receiving? *(1 choice only from a list)*

1. Raw data
2. Summary in the form of tables or graphical data
3. Automatic alerts
4. Data interpreted by another health professional
5. Other
6. None

15 What time would be ready to spend analyzing the data from connected devices each day?

O None daily
O 0-5 min
O 6-10 min
O 11-30 min
O 31-60 min
O > 60 min

16 If the device generates an alert, in what form would you prefer receiving it? *(1 choice only)*

O A call from a company
O An automatic telephone call
O An email
O A mobile text
O Other: …………

17 If you were receiving alerts, under which maximum period would you consider you had to answer the alert?

○ Immediately
○ In the hour
○ In the half day
○ In the day
○ In the week
○ You shouldn't be constrained by a period
○ Other : …………

18 Who, according to you, should be receiving the data from connected medical devices? *(1 answer per lign)*

	Yes	No	Indifferent
The general practitioner	○	○	○
The specialist of the field concerned by the data	○	○	○
The patient's pharmacist	○	○	○
The patient's nurse	○	○	○
A private enterprise	○	○	○
The patient's general health insurance	○	○	○
The patients complimentary health insurance	○	○	○
A dedicated health network	○	○	○
The patient himself	○	○	○
A helper (family member, friend…)	○	○	○
Other	○	○	○

To know you better...

19 Are you currently using a smartphone and/or a tablet?

○ Yes ○ No

If Yes to question 19:

Which one(s)? *(Multiple choice)*

☐ iPhone smartphone
☐ iPad tablet
☐ Android smartphone
☐ Android tablet
☐ Blackberry smartphone
☐ Windows smartphone
☐ Windows tablet
☐ Other : …………

20 Since when have you had your diploma in General Practice *(1 choice only from a list)*

1. < 1 year
2. 1 to 2 years
3. 2 to 5 years
4. 6 to 10 years
5. 11 to 20 years
6. 21 to 30 years
7. > 30 years

21 Are you an established general practitioner? *(1 choice only from a list)*

1. I have my own practice
2. I'm a substitute
3. I'm a salaried employee
4. I have a mixed practice

22 Do you have a teaching position in general practice at the university?

○ Yes: you are a professor, an assistant, a conference teacher, a teaching assistant

○ No

23 What is your age? *(1 choice only from a list)*

1. < 35 years-old
2. 36 to 50 years-old
3. 51 to 65 years-old
4. 66 to 80 years-old
5. > 81 years-old

24 What is your gender?

1. Female
2. Male

Do you have additional remarks on this survey or the study?

For example, would you like to specify the answers to the questions "other"? Would you like detail your vision of the future use of connected health devices in general practice?...

- Free answer

Thank you for your answers and your patience.
